# The Role of Cardiopulmonary Exercise Test in IPF Prognosis

**DOI:** 10.1155/2013/514817

**Published:** 2013-10-31

**Authors:** Christina Triantafillidou, Effrosyni Manali, Panagiotis Lyberopoulos, Likourgos Kolilekas, Konstantinos Kagouridis, Sotirios Gyftopoulos, Konstantinos Vougas, Anastasia Kotanidou, Manos Alchanatis, Anna Karakatsani, Spyros A. Papiris

**Affiliations:** ^1^2nd Pulmonary Department, “Attikon” University Hospital, Athens Medical School, National and Kapodistrian University of Athens, 1 Rimini Street, 12462 Haidari, Greece; ^2^6th Pulmonary Department, “Sotiria” Chest Diseases Hospital, 152 Mesogion Street, 11527 Athens, Greece; ^3^7th Pulmonary Department, “Sotiria” Chest Diseases Hospital, 152 Mesogion Street, 11527 Athens, Greece; ^4^Genomics and Proteomics Research Units, Center of Basic Research II, Biomedical Research Foundation, Academy of Athens, 11527 Athens, Greece; ^5^Applied Biochemical Research and Training Laboratory “Marianthi Simou”, Thorax Foundation and Department of Critical Care and Pulmonary Services, National and Kapodistrian University of Athens, “Evangelismos” Hospital, 3 Ploutarchou Street, 10675 Athens, Greece; ^6^1st Pulmonary Department, “Sotiria” Chest Diseases Hospital, Athens Medical School, National and Kapodistrian University of Athens, 152 Mesogion Street, 11527 Athens, Greece

## Abstract

*Background*. In IPF, defects in lung mechanics and gas exchange manifest with exercise limitation due to dyspnea, the most prominent and disabling symptom. *Aim*. To evaluate the role of exercise testing through the 6MWT (6-minute walk test) and CPET (cardiopulmonary exercise testing) in the survival of patients with IPF. *Methods*. This is a prospective, observational study evaluating in 25 patients the relationship between exercise variables through both the 6MWT and CPET and survival. *Results*. By the end of the observational period 17 patients were alive (33% mortality). Observation ranged from 9 to 64 months. VE/VCO_2_ slope (slope of relation between minute ventilation and CO_2_ production), VO_2_ peak/kg (peak oxygen consumption/kg), VE/VCO_2_ ratio at anaerobic threshold, 6MWT distance, desaturation, and DLCO% were significant predictors of survival while VE/VCO_2_ slope and VO_2_ peak/kg had the strongest correlation with outcome. The optimal model for mortality risk estimation was VO_2_ peak/kg + DLCO% combined. Furthermore, VE/VCO_2_ slope and VO_2_ peak/kg were correlated with distance and desaturation during the 6MWT. *Conclusion*. The integration of oxygen consumption and diffusing capacity proved to be a reliable predictor of survival because both variables reflect major underlying physiologic determinants of exercise limitation.

## 1. Introduction

Idiopathic pulmonary fibrosis (IPF) is an irreversibly progressive lung disease with substantial morbidity and mortality. No effective pharmacological treatment has been established so far [1]. Nonetheless, as research concerning IPF develops, we realize the heterogeneity of this disease which defines final prognosis [1]. Several retrospective longitudinal studies suggest a median survival time of 2 to 3 years from the time of diagnosis; nevertheless, more recent data suggest that this could be an underestimate [2]. Thus, defining prognosis remains difficult but it is of critical clinical importance.

Many prognostic factors of disease severity and outcome have been studied in IPF, either at baseline or at serial measurements over time [3]. Clinical predictors include age, gender, smoking status, dyspnea, pulmonary hypertension, and comorbidities such as emphysema [2, 4–7]. Imaging studies have shown that the overall extent of fibrosis on high-resolution computerized tomography also correlates with survival [2, 8]. Regarding pathologic predictors, the fibroblastic foci profusion has been shown in some studies to predict survival while not in others [9, 10]. Physiologic indices as predictors of survival are more extensively studied, especially the variables of the pulmonary function tests (PFTs). The forced vital capacity (FVC), total lung capacity (TLC), and diffusion capacity for carbon monoxide (DLCO) have consistently proved to relate with survival, while 6- and 12-month changes in FVC and DLCO are found even more reliable in estimating survival [11, 12].

In IPF, anatomic and functional derangements lead to defects in lung mechanics and gas exchange clinically manifesting with exertional dyspnea, the most prominent and disabling symptom in these patients [13]. Exercise limitation has a tremendous negative impact on the quality of life of IPF patients [14], and one would expect that it would additionally inversely influence their outcome. However, exercise testing in the staging and prognosis of the disease is less extensively studied than resting variables [15]. More precisely, the 6-minute walking test (6MWT), a practical and simple test which reflects submaximal exercise, has only recently been included in the evaluation of patients with IPF [16]. Desaturation and distance walked during the 6MWT have proved valuable in the estimation of prognosis [17, 18].

 Cardiopulmonary exercise test (CPET) is a dynamic, accurate, and reliable tool for the estimation of severity and prognosis for patients with cardiovascular and respiratory diseases reflecting the abnormalities of both systems under submaximal and peak exercise performance [19]. However, it is considered to add little to resting lung function in assessing the severity and outcome in interstitial lung diseases [15, 16]. 

Based on our recent study showing significant associations between clinical predictors of survival such as the Medical Research Council (MRC) chronic dyspnea score and physiological variables obtained during maximal and submaximal exercise testing in IPF patients [13], we attempted in the present study to evaluate the role of exercise testing through two of the most commonly used exercise protocols, the 6MWT and CPET as a prognostic tool in IPF. 

## 2. Materials and Methods

### 2.1. Study Subjects and Setting

This observational study was approved by the Institutional Ethics Committee of “Attikon” University Hospital, National and Kapodistrian University of Athens, Greece. Written informed consent was obtained from each patient. We prospectively recruited patients examined at the outpatient clinic over a period of one year. All patients fulfilled the criteria of the American Thoracic Society, European Respiratory Society, and the American College of Chest Physicians for the diagnosis of IPF [20]. These patients have already been recruited in another study by our team [13]. After completion of the study all patients were reexamined as far as diagnosis was concerned based on the recently published criteria of 2011 [1]. All patients were found to fulfill the new criteria of IPF. None of the patients were receiving treatment with oxygen. None of the patients included had significant pulmonary hypertension (PASP > 45 mmHg, verified by cardiac ultrasound). Patients taking beta-blockers were specifically excluded from performing the CPET and by extension from the study. No case of lung transplant was recorded during the study. Secondary causes of lung fibrosis were excluded: none of the patients had a history of environmental or occupational exposure, drug toxicity, or autoimmune rheumatic disease, as documented by history, clinical and immunological tests.

### 2.2. Pulmonary Function Tests (PFTs)

Lung function tests were done at diagnosis or at an interval of 15 days before the realization of the 6MWT and the CPET. PFTs included forced expiratory volume during the first second of expiration (FEV_1_), forced vital capacity (FVC), total lung capacity (TLC), and single-breath carbon monoxide diffusing capacity (DLCO) all measured by MasterScreen Body apparatus (Erich Jaeger GmbH, Wuerzburg, Germany).

### 2.3. The 6-Minute Walking Test

The 6MWT was done according to the ATS guidelines [21]. The following data were collected and analyzed: distance (meters), duration (minutes), oxygen saturation at the initiation and at the end of the test, pulse at initiation and at the end of the test, the difference in oxygen saturation before and after the test, systolic and diastolic blood pressure before the test, Borg scale score before and after the test, and age (years), height (meters), and body weight of the patients (kilograms). Oxygen desaturation was defined as resting oxygen saturation minus oxygen saturation at the end of the 6MWT.

### 2.4. Cardiopulmonary Exercise Testing

The CPET was performed at an interval of 2-3 days after the 6MWT using a standardized protocol in accordance with the American Thoracic Society/American College of Chest Physicians (ATS/ACCP) statement [19]. All patients underwent a symptom-limited cardiopulmonary exercise test with an electromagnetically braked cycle ergometer (Ergometrics 900, Erich Jaeger GmbH, Wurzburg, Germany) using a ramp protocol. The protocol included 3 min of sitting rest, 3 min of unloaded cycling (at 60 revolutions per min plus or minus 5 revolutions per min), followed by a progressively increasing work rate in a ramp fashion, and 3 min of recovery. The work rate increment for each ramped exercise test was individualized on the basis of each patient's pretest activity level (range, 8 to 25 Watts/min). Cardiopulmonary data were collected and analyzed with an exercise metabolic unit (Oxycon Pro Erich Jaeger GmbH, Wurzburg, Germany). The following parameters were recorded: heart rate (HR), minute ventilation (VE), tidal volume (TV), peak oxygen consumption (VO_2_ peak), peak oxygen consumption/kg (VO_2_ peak/kg), %VO_2_ predicted, slope of relation between minute ventilation and CO_2_ production (VE/VCO_2_ slope), VE/VCO_2_ ratio at anaerobic threshold, respiratory rate (RR), oxygen pulse (O_2_P), oxygen saturation at peak exercise (SpO_2_ peak), anaerobic threshold (AT), breathing reserve (BR), heart rate recovery (HRR), and heart rate reserve (HRRes). AT was determined noninvasively through the plot of VCO_2_ versus VO_2_ (V-slope method).

### 2.5. Statistical Analysis

Data are presented as mean ± standard deviation (±SD). The parameters of the current study were individually evaluated for relevance to the overall survival through the Cox proportional hazards models. It must be noted that among parameters which presented an absolute correlation value greater than 90%, only one was included in the evaluation. Each of these models was evaluated by three independent and asymptotically equivalent tests, the Wald test, the likelihood ratio test, and the score (log rank) test, each calculating a *P* value for the null-hypothesis that the factor coefficients are equal to zero. For evaluating the models and ranking them from best to worst under the frame of the aforementioned tests, a sum-index was calculated by summing the *P* values of the aforementioned tests and the following selection criteria for the best model were set.

1. The best model should minimize the sum-index.
2. The null-hypothesis should be rejected by as many of the individual tests as possible after applying the Bonferroni correction for multiple testing which set the a-level of statistical significance at 0.0019; hence, only *P* values lower than that threshold were considered statistically significant.

In order to optimally model the risk through multiple regression hazard modelling, all the parameters having the null-hypothesis rejected in at least one of the tests after the Bonferroni correction were included in a multiple regression Cox proportional hazards model, and the optimum model was selected through a stepwise model selection procedure. Specifically the algorithm utilized for the stepwise procedure was bidirectional (combined forward selection and backward elimination) aiming at minimizing the Bayesian information criterion (BIC) [22] which is a robust measure of model adequacy. Statistical analysis was carried out using R. The Cox proportional hazards modelling was performed utilizing the “Survival” R-package. The stepwise model selection procedure was implemented by the “MASS” R-package. The nonparametric Spearman correlation coefficient was calculated to describe the relationships between variables of 6MWT, CPET, and pulmonary function tests by using SPSS v.13.0.0 (Chicago, IL). A *P* value less than 0.05 was considered significant. 

## 3. Results

**Figure 1:**
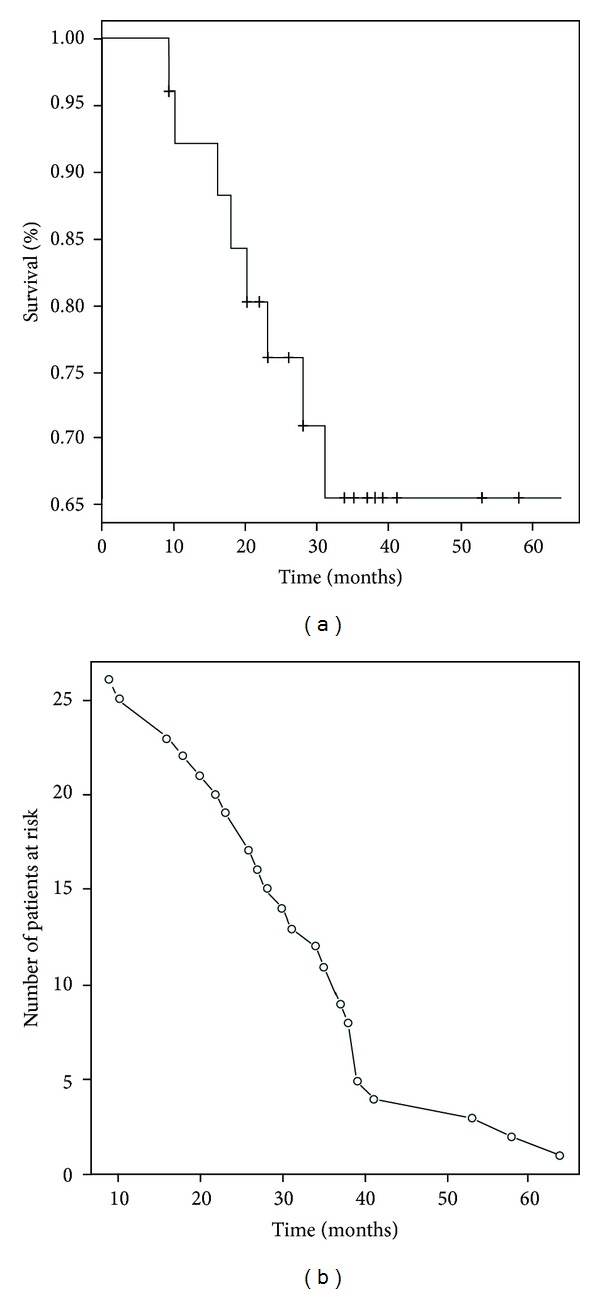
The cumulative Kaplan-Meier survival plot. (a) Survival of 25 IPF patients followed till death (uncensored: *n* = 8) or reporting of the study (censored: *n* = 17), combined by a plot (b) where the number of patients at risk is shown.

**Figure 2:**
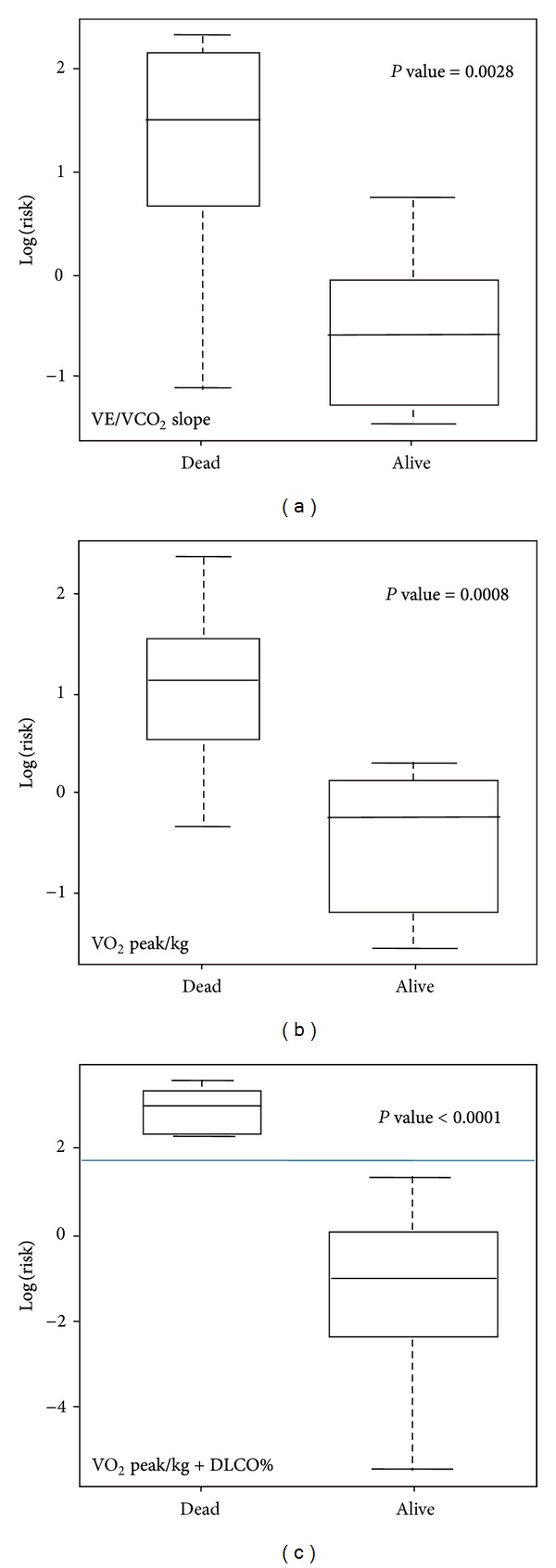
Overall death risk through the Cox proportional hazards models. In box plot (a), the Cox model includes only VE/VCO_2_ slope, in box plot (b), the Cox model includes VO_2_ peak/kg, and in box plot (c), the Cox model includes VO_2_ peak/kg + DLCO%. A bidirectional stepwise model selection method minimizing the Bayesian information criterion (BIC) was utilized for selecting the optimal model which was identified as VO_2_ peak/kg + DLCO%. Data are described using standard box plots with medians (interquartile range). Risk was found to be significantly differentiated between dead and alive with *P* = 0.0028, *P* = 0.008, and *P* < 0.0001, respectively.

**Figure 3:**
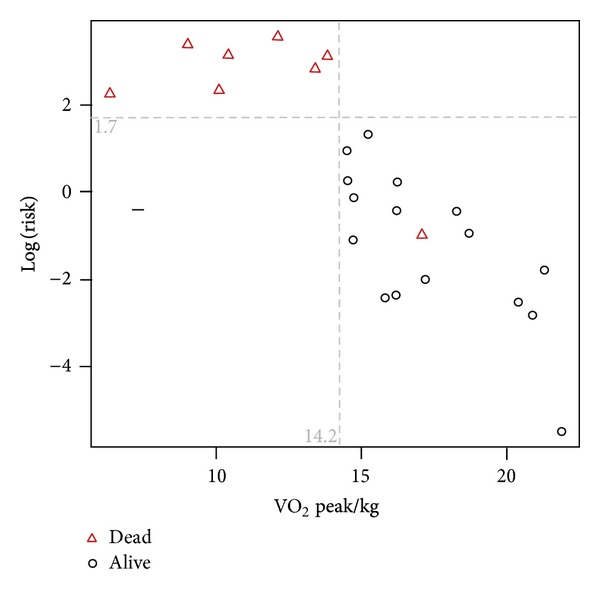
A threshold of mortality was identified. A threshold VO_2_ peak/kg of 14.2 mL/min/kg was associated with an increased risk of mortality according to optimum Cox proportional hazards model.

The population studied consisted of 25 patients. The demographic and clinical characteristics of the study population at the time of IPF diagnosis are given in Table 1. All patients were treatment naive when entering the study, while most of them received no treatment for IPF in the observational period as well. None of the patients included in the study were on treatment with beta-blockers. At the time of reporting seventeen patients were still alive (Figure 1). Follow-up time ranged from 9 months to 64 months. Mean survival was 48.7 ± 4.4 months (95% CI = 40–57.4) (Figure 1). All deaths were disease related. In Table 2, the results are reported for the 6MWT and the CPET. Out of 25 patients, 16 patients (64%) stopped CPET due to dyspnea and the rest 9 (36%) due to leg discomfort. Twenty-one patients reached anaerobic threshold. Among the 4 patients who did not reach AT, one stopped due to leg discomfort and the rest due to dyspnea. Among CPET parameters, VE/VCO_2_ slope, VO_2_ peak/kg, and VE/VCO_2_ at anaerobic threshold were significant predictors of survival (Table 3). As far as 6MWT is concerned, distance walked and oxygen desaturation were significant predictors of survival, while among PFTs parameters, only DLCO% correlated with survival (Table 3). Among all parameters, VE/VCO_2_ slope and VO_2_ peak/kg were the factors with the two smallest sum-indexes which reject the null-hypothesis in all the independent tests after the Bonferroni correction; thus, they were the most significant parameters predicting survival. These factors present a negative correlation of 76%. After further analysis with the stepwise model selection, the optimal model for survival prediction was identified as VO_2_ peak/kg + DLCO% (Figure 2). Per 1-unit increase of VO_2_ peak/kg (1 mL/kg/min) and DLCO% (1%), mortality risk is reduced by 32% and 13%, respectively (95% CI 0.60–0.95, HR = 0.75 for VO_2_ peak/kg, 95% CI 0.80–0.96, HR = 0.88 for DLCO%). Regarding “Distance” which was the only statistically significant value of the 6MWT (after the Bonferroni correction), it must be noted that it was included in the initial model being evaluated by the stepwise model selection procedure minimizing the BIC and it was rejected along the process. Additionally, it is worth mentioning that when including the Distance in a Cox proportional hazards model, the R-squared of this model is 0.376, while the respective R-squared of the optimum model as evaluated by the stepwise model selection procedure was 0.528 which is a clear indication that the optimum model provides a more efficient modeling of the hazard ratios than the model involving the Distance 6MWT parameter. Furthermore, according to optimum Cox proportional hazards model, a threshold VO_2_ peak/kg of 14.2 mL/min/kg was associated with an increased risk of mortality (Figure 3). VE/VCO_2_ slope and VO_2_ peak/kg were also found to correlate with distance and desaturation during the 6MWT (Table 4). VO_2_ peak/kg, distance walked, and desaturation correlated with resting functional variables such as the FEV_1_, FVC, TLC, and DLCO, while VE/VCO_2_ slope correlated only with DLCO (Table 5). 

## 4. Discussion

In this study, we aimed to evaluate the role of exercise testing in the outcome of IPF patients. Therefore, we examined the relationship between the variables of maximal-CPET and submaximal-6MWT exercise tests and survival. We found that VE/VCO_2_ slope, VO_2_ peak/kg, and VE/VCO_2_ ratio at anaerobic threshold from the CPET and both distance walked and desaturation from the 6MWT are significantly correlated with survival. According to our results, the variables most strongly correlated with survival are VE/VCO_2_ slope and VO_2_ peak/kg, both from the CPET protocol, while the optimal model for mortality risk estimation was identified when VO_2_ peak/kg and DLCO% were combined. In this model, a threshold VO_2_ peak/kg of 14.2 mL/min/kg was associated with an increased risk of mortality. 

The results of the present study are partly in accordance with the ones of Fell and colleagues who demonstrated that cardiopulmonary exercise testing adds significant prognostic information for patients with IPF and identified that IPF patients with a baseline maximal oxygen uptake less than 8.3 mL/kg/min during CPET have an increased risk of death. VO_2_ peak/kg did not correlate with survival when examined as a continuous variable and the baseline VO_2_ peak/kg threshold with a predictive role was lower compared to the one found in the present study [23]. In addition, in their study they hypothesized that short-term longitudinal changes in VO_2_ peak/kg would predict survival, but according to their results, VO_2_ peak/kg did not change between baseline and 6 months. The discrepancies in the values and thresholds of the significant variables such as VO_2_ peak/kg could be attributed to differences in the characteristics of the studies' populations and do not alter the similarities in the conclusions and the common clinical implication of both studies. For example, patients in the study of Fell and colleagues who were recruited from previous study protocols have a more impaired functional status as expressed by FVC values and were under treatment with several regimens contrary to the present study where most patients are treatment naive. In a recent study which retrospectively examined the prognostic value of CPET in 63 patients with IPF, results showed that increased ventilation at anaerobic threshold (AT) as reflected by higher VE/VO_2_ at AT was a major prognostic factor of death [24]. On the other hand, in the study of Gay and coworkers, VO_2_ peak measurement among the factors of a composite clinical, radiographic, and physiologic scoring system for IPF patients failed to predict survival [25], while Erbes and colleagues found that gas transfer during spiroergometric exercise was not predictive of prognosis in IPF [26]. However, most of those studies were retrospective in design.

The significant correlation of CPET variables with survival indicates that exercise limitation has a tremendous effect on this group of patients. In IPF, fibrotic lung parenchymal damage leads to multiple physiologic derangements such as low tidal volume, the rapid shallow breathing pattern, and the detrimental dead space ventilation. The above disorders result in worsening gas exchange during exercise sooner or later in every patient with IPF. Exercise limitation is further aggravated by pulmonary vascular derangements, myocardial disturbances, and peripheral muscle weakness that are commonly described in IPF patients [13]. Based on the results of the present study, the physiological factors that have the most potent impact on survival are VE/VCO_2_ slope and VO_2_ peak/kg. In patients with chronic lung diseases, an elevated VE/VCO_2_ slope is attributed to the effects of increased physiological dead space, ventilation-perfusion mismatching, and the abnormally elevated chemoreceptor and ergoreceptor sensitivity that are present at rest and deteriorate during exercise [27]. Furthermore, pulmonary hypertension that commonly develops in IPF patients has been shown to be associated with an elevated VE/VCO_2_ slope [27]. In many studies, VE/VCO_2_ slope has been shown to have similar or even superior ability over VO_2_ peak/kg to predict serious cardiovascular events in heart failure, the disease where CPET has gained widespread use and most clinical experience exists [28]. 

Based on our results, the predictive role of CPET and more precisely of VO_2_ peak/kg is further enforced when the model combines VO_2_ peak/kg with DLCO. This finding could be explained by the fact that both values reflect very robustly and in a complementary way the pathophysiology of exercise limitation in IPF patients. Peak oxygen consumption on the one hand reflects the attainment of a limitation at some point in the oxygen conductance pathway due to the physiologic derangements already described in IPF. On the other hand, DLCO provides information regarding the above-mentioned physiologic derangements, that is, the gas transfer capability which is significantly impaired in IPF and predicts oxygen desaturation during exercise [11, 12, 29]. 

The evaluation of multidimensional models of survival, combining variables that independently predict mortality and share common underlying physiologic determinants, has already been studied in respiratory diseases such as COPD [30]. In IPF, most composite indices that have been identified by now usually derive from the extent of fibrosis on HRCT, pulmonary function test variables, and clinical parameters such as age and gender, thus precluding the prognostic strength of exercise data [12, 31, 32]. Only in the study of King Jr. and coworkers was PaO2 at the end of maximal exercise included among seven parameters in the new complete clinical, radiographic, and physiologic (CRP) scoring system for prediction of survival in IPF patients [12]. Nevertheless, this score is not easy to perform in everyday clinical practice and it partially depends on subjective measurements such as finger clubbing. 

As far as the contribution of the 6MWT in the prognostic evaluation of IPF patients is concerned, the present data are in agreement with the conclusions of previous studies showing that a decrease in saturation of 4% or oxygen desaturation below 88% during 6MWT is a predictor of mortality for IPF patients [17]. The role of the distance walked is also highlighted, although contradictory conclusions exist on the field [18, 33]. However, the predictive role of the 6MWT seems weaker when compared to CPET when multivariable relationships are explored. This observation is in line with the conclusion of Fell and coworkers that VO_2_ peak/kg threshold is a more robust predictor of survival than desaturation beyond 88% during a 6MWT [23]. Based on the results of both studies, we believe that the role of CPET in the evaluation of survival in IPF patients could be upgraded. CPET should be applied in a complementary way to the 6MWT which is much more widely performed and recommended [16].

In the present study, further evaluation of the interdependence of both maximal and submaximal exercise test variables with resting functional measurements has demonstrated a strong and significant association between them. The fact that all four pulmonary function test parameters at rest (FEV_1_, FVC, TLC, and DLCO) were found to correlate significantly with indices of both 6MWT and CPET indicates that the group of patients is well selected. The restrictive defect encountered in this group of patients leads to the inability to expand tidal volume appropriately and therefore to low breathing reserve during the increased metabolic demand of exercise which is a major determinant of exercise limitation in IPF [29, 34]. 

Our study has a number of limitations, the most important being the relatively short observation period and in consequence the limited mortality rate. Another limitation is the moderate number of patients included mostly due to the rarity of the disease with a prevalence of 3.4 cases per 100.000 inhabitants in Greece [35]. However, it is a prospective, single centre study based on a well-selected group of IPF patients most of who are treatment naive, in contrast to the majority of already existing studies in the literature which are retrospective in design and examine patients that participate in various treatment protocols that could have an undefined impact on their survival. 

## 5. Conclusions

In conclusion, exercise testing through two of the most commonly used protocols, the 6MWT and CPET, is shown to have a significant prognostic role for the survival of IPF patients. Most importantly, variables of cardiopulmonary exercise testing such as VE/VCO_2_ slope and VO_2_ peak/kg are found to be more potent than previously thought concerning the prediction of outcome in IPF, not only as thresholds but also as continuous variables. The predictive role of peak oxygen consumption is further reinforced when integrated with DLCO due to the fact that both variables share major underlying physiologic determinants of exercise limitation in IPF.

## Figures and Tables

**Table 1:** Demographic and clinical data of the study population.

Variables (*n*)	(mean ± SD)
Age, year (*n* = 25)	67.5 (±8.3)
Gender (M/F)	17/8
Ex-smoker (*n*) %	13 (52%)
Non smoker (*n*) %	11 (44%)
Smoker (*n*) %	1 (4%)
PY (mean ± SD)	25.4 (±34.2)
FEV_1_% (*n* = 25)	80.4 ± 18.8
FVC% (*n* = 25)	77.5 ± 21.8
FEV_1_%/FVC (*n* = 25)	82 ± 0.04
TLC% (*n* = 23)	61.4 ± 13.7
DLCO% (*n* = 23)	45.6 ± 13.2
Comorbid disease (*n* = 25)	
Arterial hypertension	10 (40%)
Coronary disease	4 (16%)
Diabetes mellitus	3 (12%)
Gastroesophageal reflux symptoms	5 (20%)

M/F: male/female, PY: pack/years, FEV_1_: forced expiratory volume at one second, FVC: forced vital capacity, TLC: total lung capacity, and DLCO: diffusing capacity for carbon monoxide. All values are shown as mean ± standard deviation (SD).

**Table 2:** Results of the CPET and the 6MWT in the study population.

	*n*	Mean ± SD
CPET variable		
VO_2_ peak/kg (mL/kg/min)	25	15.5 ± 3.9
VO_2_ at AT (mL/kg/min)	21	11.6 ± 3.5
Oxygen pulse (mL/beat)	25	9.2 ± 2.8
SpO_2_ peak	25	87.7 ± 5.7
VE peak (L/min)	25	54.2 ± 17.5
BR peak %	25	27.3 ± 21.2
VE/VCO_2_ slope	25	40.0 ± 13.5
VE/VCO_2_ ratio at AT	21	37 ± 10.4
HR reserve	25	19.2 ± 18.0
HR recovery	25	14.5 ± 8.3
6MWT variable		
Distance (meters)	25	326.4 ± 153
SpO_2_ at the initiation%	25	94.8 ± 2.2
SpO_2_ at the end%	25	87.6 ± 5.6
Desaturation (%)	25	7.2 ± 4.3

Peak oxygen consumption/kg (VO_2_ peak/kg), anaerobic threshold (AT), oxygen pulse (O_2_P), oxygen saturation at peak exercise (SpO2 peak), total ventilation (VE), carbon dioxide output (VCO_2_), breathing reserve (BR), VE/VCO_2_ slope at anaerobic threshold (AT), and heart rate (HR). All values are shown as mean ± standard deviation.

**Table 3:** Significant predictors of survival among the variables of CPET and 6MWT in IPF patients.

Variables	Wald test	Score (log rank) test	HR	CI (95%)	Sum-index
VE/VCO_2_ slope	0.001	0.0002	1.09	1.04–1.15	0.0017
VO_2_ peak/kg	0.001	0.0004	0.75	0.60–0.95	0.0033
DLCO%	0.002	0.0007	0.88	0.80–0.96	0.0035
Distance (meters)	0.003	0.0008	0.99	0.98–1.00	0.0047
VE/VCO_2_ ratio at AT	0.006	0.0001	1.15	1.04–1.26	0.0074
Desaturation (%)	0.007	0.0025	1.45	1.11–1.90	0.0102

Minute ventilation (VE), carbon dioxide output (VCO_2_), peak oxygen consumption/kg (VO_2_ peak/kg), diffusing capacity for carbon monoxide% (DLCO%), VE/VCO_2 _slope at anaerobic threshold (AT), and hazard ratio (HR).

**Table 4:** Relationships between the variables of 6MWT and VE/VCO_2_ slope and VO_2_ peak/kg by Spearman's rank correlation coefficient. *P* value < 0.05 is considered significant.

	VO_2_ peak/kg	VE/VCO_2_ slope
Distance	*r* = 0.7	*r* = −0.6
	*P* < 0.0001	*P* = 0.001

Desaturation%	*r* = −0.52	*r* = 0.4
	*P* = 0.007	*P* = 0.02

Minute ventilation (VE), carbon dioxide output (VCO_2_), and peak oxygen consumption/kg (VO_2_ peak/kg).

**Table 5:** Relationships between the variables of both exercise tests and pulmonary function tests in the study population by Spearman's rank correlation coefficient. *P* value < 0.05 is considered significant.

	VE/VCO_2_ slope	VO_2_ peak/kg	Distance 6MWT	Desaturation 6MWT
FEV_1_%	*r* = −0.26	*r* = 0.41	*r* = 0.4	*r* = −0.47
	*P* = 0.2	*P* = 0.03	*P* = 0.05	*P* = 0.01

FVC%	*r* = −0.38	*r* = 0.52	*r* = 0.48	*r* = −0.5
	*P* = 0.06	*P* = 0.007	*P* = 0.01	*P* = 0.01

TLC%	*r* = −0.36	*r* = 0.6	*r* = 0.61	*r* = −0.47
	*P* = 0.08	*P* = 0.003	*P* = 0.002	*P* = 0.02

DLCO%	*r* = −0.74	*r* = 0.64	*r* = 0.7	*r* = −0.57
	*P* < 0.001	*P* = 0.001	*P* < 0.0001	*P* = 0.004

Minute ventilation (VE), carbon dioxide output (VCO_2_), peak oxygen consumption/kg (VO_2_ peak/kg), (FEV_1_) forced expiratory volume at one second, (FVC) forced vital capacity, (TLC) total lung capacity, and (DLCO) diffusing capacity for carbon monoxide.
